# Identification of a Novel Glycosyltransferase Prognostic Signature in Hepatocellular Carcinoma Based on LASSO Algorithm

**DOI:** 10.3389/fgene.2022.823728

**Published:** 2022-03-09

**Authors:** Zhiyang Zhou, Tao Wang, Yao Du, Junping Deng, Ge Gao, Jiangnan Zhang

**Affiliations:** ^1^ Department of General Surgery, The First Affiliated Hospital of Nanchang University, Nanchang, China; ^2^ Department of Day Ward, The First Affiliated Hospital of Nanchang University, Nanchang, China

**Keywords:** glycosyltransferase, hepatocellular carcinoma, overall survival, prognostic signature, lasso regression analysis

## Abstract

Although many prognostic models have been developed to help determine personalized prognoses and treatments, the predictive efficiency of these prognostic models in hepatocellular carcinoma (HCC), which is a highly heterogeneous malignancy, is less than ideal. Recently, aberrant glycosylation has been demonstrated to universally participate in tumour initiation and progression, suggesting that dysregulation of glycosyltransferases can serve as novel cancer biomarkers. In this study, a total of 568 RNA-sequencing datasets of HCC from the TCGA database and ICGC database were analysed and integrated via bioinformatic methods. LASSO regression analysis was applied to construct a prognostic signature. Kaplan–Meier survival, ROC curve, nomogram, and univariate and multivariate Cox regression analyses were performed to assess the predictive efficiency of the prognostic signature. GSEA and the “CIBERSORT” R package were utilized to further discover the potential biological mechanism of the prognostic signature. Meanwhile, the differential expression of the prognostic signature was verified by western blot, qRT–PCR and immunohistochemical staining derived from the HPA. Ultimately, we constructed a prognostic signature in HCC based on a combination of six glycosyltransferases, whose prognostic value was evaluated and validated successfully in the testing cohort and the validation cohort. The prognostic signature was identified as an independent unfavourable prognostic factor for OS, and a nomogram including the risk score was established and showed the good performance in predicting OS. Further analysis of the underlying mechanism revealed that the prognostic signature may be potentially associated with metabolic disorders and tumour-infiltrating immune cells.

## Introduction

Hepatocellular carcinoma (HCC) is a highly aggressive solid malignancy and the fourth leading cause of cancer-related death, which imposes a tremendous health and socioeconomic burden globally (Singal, et al., 2020). Studies have shown that hepatitis virus infection, alcohol-related liver disease (ALD), non-alcoholic fatty liver disease (NAFLD) and non-alcoholic liver steatohepatitis (NASH) are the main aetiological risk factors for the development of HCC. Chronic infections with hepatitis virus are still the strongest risk factors for HCC in developing countries, nevertheless, NAFLD is gradually becoming the leading cause of HCC in Western countries (Huang, et al., 2021a). Despite all efforts made in the past to improve the prognosis of HCC, the prognosis remains poor, with an overall 5-years survival rate of approximately 18%, which is only slightly better than that of pancreatic cancer (Jemal, et al., 2017).

Of note, the prediction of clinical outcomes provides vital and necessary medical information. The traditional TNM staging system, which mainly relies on clinicopathological parameters, cannot provide a precise prediction of prognosis in clinical practice. In particular, HCC is a malignant tumour with the characteristic of high heterogeneity, which adds to the complexity of accurately predicting prognosis. One possible strategy to improve predictive outcome is to better understand the fundamental biological processes of cancer cells, and to identify prognostic signatures to stratify patients for individualized precision therapies based on prognosis and metastatic potential.

Glycosylation, the most universal protein post-translational modification, is an enzymatic process that catalyses the transfer of carbohydrate chains to proteins by glycosyltransferases (GTs) and glycosidases (Pinho and Reis, 2015). So far, 14 distinct human protein glycosylation pathways have been outlined, which are directed by at least 173 different GTs (Schjoldager, et al., 2020). They are divided into four main types: N- glycosylation, O- glycosylation, C-mannosylation and glypiation. Modified proteins are involved in nearly all biological processes, especially intercellular signal transduction and the immune response (Johannssen and Lepenies, 2017; Indellicato and Trinchera, 2021).

Alterations in cellular glycosylation have been recognized as hallmarks of malignant tumours, which contribute to sustaining proliferative signalling and metabolism, promoting invasion and metastasis, and immune evasion (Munkley and Elliott, 2016; Thomas, et al., 2021; Dobie and Skropeta, 2021; Rodrigues, et al., 2021). The under- or overexpression of GTs is the main contributor to cancer initiation and progression. Fucosylation is one of the most common modifications in the glycosylation pattern of HCC (Zhang, et al., 2017). The core fucosylation of *α*-fetoprotein (AFP-L3), a typical modified product, has already been confirmed as a biomarker in detecting early HCC (Wu, et al., 2014a; Noda, et al., 1998). As key enzymes of fucosylation, FUT1 (Kuo, et al., 2017), FUT8 (Cheng, et al., 2016) and POFUT1 (Ma, et al., 2016) are highly expressed and positively associated with advanced stage and poor prognosis in HCC. Other members of the FUT family, such as FUT2 (Wu, et al., 2014b), FUT4 (Cheng, et al., 2013), FUT6 (Guo, et al., 2012), and FUT7 (Wang, et al., 2005), are also known to support the development of HCC. Similarly, aberrant O-GlcNAcylation due to dysregulation of O-linked N-acetylglucosamine (GlcNAc) transferase (OGT) expression has been shown in HCC (Makwana, et al., 2019). OGT can promote migration by regulating FOXA2 stability and transcriptional activity (Huang, et al., 2021b), and the stem-like cell potential through O-GlcNAcylation of eIF4E (Cao, et al., 2019). In addition, abnormal expression of other kinds of GTs has been described in HCC, including C1GALT1, GALNT1, GALNT2, GALNT4, MGAT4A, MGAT5, B3GALT5, B4GALT4, ST3GAL1, ST3GAL2, ST3GAL6 and ST6GAL1. Cumulative findings indicate that abnormal expression of GTs seems to be a general feature of cancer cells and contributes to tumorigenesis and additional malignant characteristics.

Given the diversity of GTs and the high heterogeneity in individuals, a comprehensive understanding of the crucial role of aberrant glycosylation in HCC progression can further provide assistance in predicting prognosis. Therefore, the development of a novel evaluation index of glycosylation may be very useful for prognosis research. In this study, we developed a 6-gene prognostic signature that focused on the prognostic value of GT in HCC and validated its predictive capability through a variety of computational approaches.

## Materials and Methods

### Data Screening and Gene Integration

Both the complete clinicopathologic information and matched RNA-sequencing FPKM data (HTSeq-FPKM) of HCC samples were extracted from The Cancer Genome Atlas (TCGA) data portal (up to September 23, 2021,https://portal.gdc.cancer.gov/). We also downloaded clinical and mRNA expression data of a Japanese cohort from the International Cancer Genome Consortium (ICGC) data portal (up to November 27,2019,https://dcc.icgc.org/).

Two glycosylation-related gene sets were obtained from the GlycoGene DataBase (GGDB: https://acgg.asia/ggdb2/index) and Hugo Gene Nomenclature Committee (HGNC: https://www.genenames.org/data/genegroup/#!/group/424). Gene set intersections were regarded as GT sets. Differentially expressed genes (DEGs) of the GT set between HCC and normal samples were identified using the “limma” package in R, and the screening criteria were FDR <0.05. Meanwhile, univariable Cox regression was employed to evaluate the association of each DEG with survival and results with a *p* value <0.05 were selected as prognosis-related genes. Finally, prognosis-related differentially expressed GTs were obtained.

### Construction and Validation of the Prognostic Signature

All incorporated HCC samples from the TCGA database were randomly assigned to training and testing cohorts at a 1:1 ratio. A prognostic signature was constructed by applying the Least Absolute Shrinkage and Selection Operator (LASSO) regression method, and the product of gene expression i and the corresponding coefficient $\beta_{i}$ of each gene were added to establish the risk score: risk score = $\overset{n}{\underset{i=1}{\sum }}\beta_{i}*i$ .

Utilizing the risk score formula, samples in the training cohort were categorized into high-risk and low-risk groups via the threshold of the median score. The Kaplan-Meier method was performed to compare survival differences between the two groups, and the prognostic value of the prognostic signature was shown by the receiver operating characteristic (ROC) curve. Simultaneously, we validated its prognostic performance with the TCGA testing cohort and ICGC external validation cohort.

### Clinicopathological Features and Development of a Nomogram

Univariate and multivariate Cox regression analyses were performed to display the prognostic performance of this signature with other clinicopathological features.

A nomogram was developed to calculate individual’s probability of overall survival (OS) by using the risk scores and clinical indicators. The final sum of the scores was expected to be the corresponding 1-, 2-, and 3-years survival probability.

### Gene Set Enrichment Analysis and Correlation of Tumour-Infiltrating Immune Cells

GSEA was performed based on the gene matrix (“c2.cp.kegg.v7.4.symbols” and “c5.go.v7.4.symbols”) between the high-risk and low-risk groups.

The CIBERSORT algorithm was used to calculate the relative abundance of 22 tumour-infiltrating immune cells in each sample of the TCGA dataset and ICGC dataset.

### Cell Culture and the Experimental Validation *in vitro*

The LO2 human hepatocyte cell line and HepG2 human hepatoma cell line were cultured in Dulbecco’s modified Eagle’s medium (DMEM, Gibco, United States) supplemented with 10% foetal bovine serum (FBS, Gibco, United States) and incubated in a humidified atmosphere at 37 °C with 5% CO2.

Total RNA was extracted by using TransZol Up (TransGen Biotech, China) following the manufacturer’s protocols. cDNA was synthesized using the PrimeScript RT reagent kit with gDNA Eraser (Takara, Japan), and mixed with primers (Supplementary Table S1) and TB Green Premix Ex Tap II (Takara, Japan), and run in the CFX96 Real-Time PCR Detection System (Bio–Rad, United States). The relative expression of the prognostic signature mRNA was calculated by the 2^−ΔΔCt^ method with GAPDH as the reference.

Total protein was prepared with RIPA buffer (Solarbio, China) with protease and phosphatase inhibitor cocktails (Solarbio, China). The protein levels were quantified by the BCA protein assay kit (Solarbio, China). Next, proteins were loaded onto 10% SDS-PAGE gel, separated electrophoretically, and transferred to PVDF membranes (Millipore, United States). After blocking with 5% non-fat milk for 1 h, the membranes were incubated at 4°C overnight with primary antibodies against POMGNT1 (Immunoway, YT6311), B4GALT3 (Immunoway, YT5009), DPM1 (Proteintech, 12403-2-AP), B4GALT2 (Proteintech, 20330-1-AP), B4GALNT1 (Proteintech, 13396-1-AP), B3GAT3 (ABclonal, A20618), and GAPDH (Immunoway, YT5052). The next day, we incubated the PVDF membranes with HRP-conjugated secondary antibodies (mouse or rabbit) at room temperature for 1 h. The immunoblot signals were visualized using the hypersensitive ECL chemiluminescence detection kit (Proteintech, PK10003).

The protein expression levels of the prognostic signature were verified between normal tissues and cancer tissues from The Human Protein Atlas (HPA: https://www.proteinatlas.org/).

### Statistical Analysis

All statistical analyses were performed with R software (version 4.0.4) and Strawberry Perl (version 5.32.0.1). LASSO regression analysis was applied to construct the prognostic signature. Nomogram construction and validation were performed using Iasonos’ guide. The survival predictive accuracy of the risk assessment model was evaluated using time-dependent ROC curve analysis. Differences with *p* < 0.05 were considered statistically significant.

## Results

### Dataset Characteristics and Candidate Gene Identification

**FIGURE 1:**
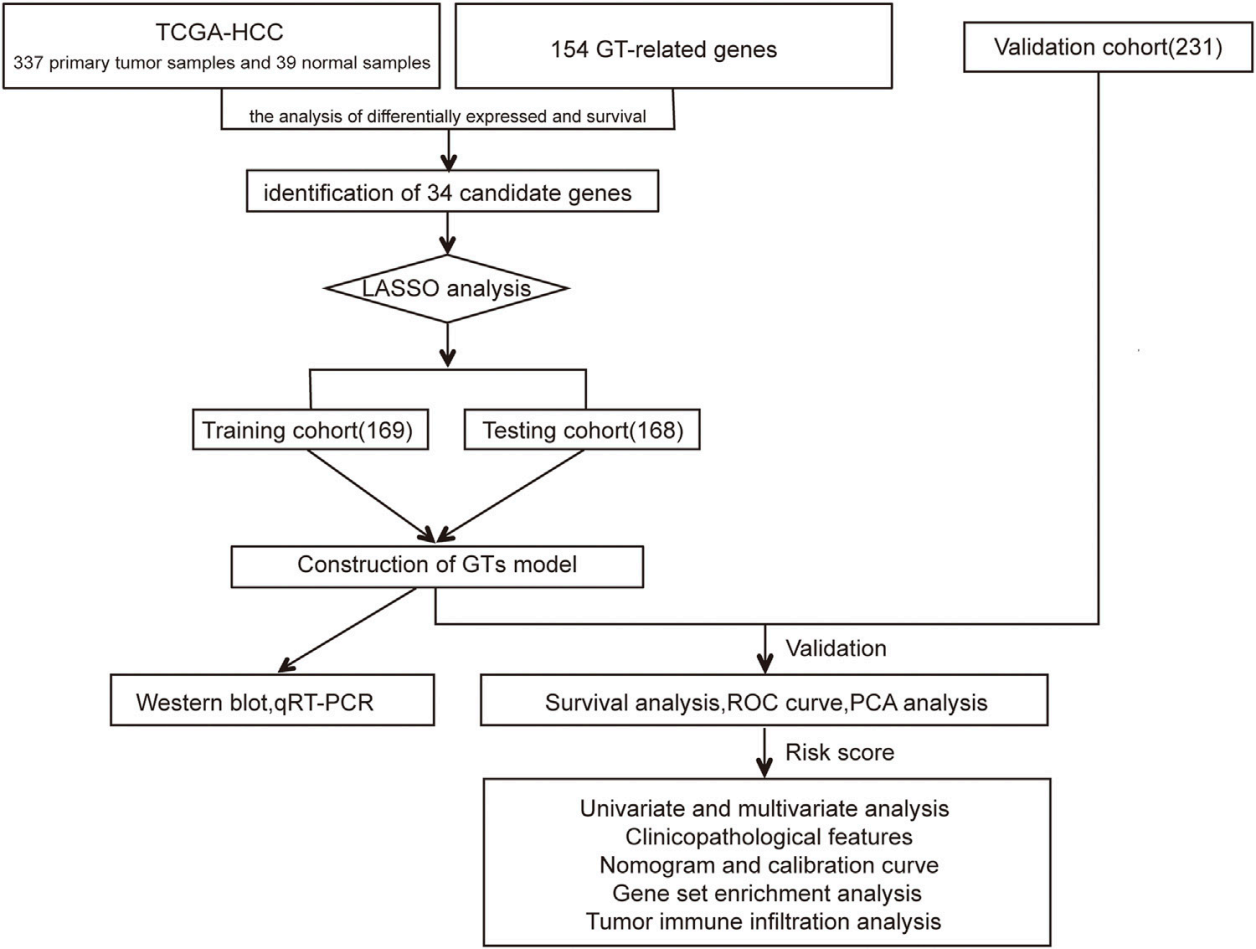
Flowchart of our study.

The flow chart of this study design is depicted in Figure 1. In the TCGA dataset, 337 primary HCC samples and 39 normal samples were screened as training cohort and testing cohort. In the ICGC dataset, a total of 231 tumour samples with HCC were available for external validation. The detailed characteristics are shown in Table 1.

**TABLE 1:** Clinical characteristics of samples involved in this study.

Characteristics	TCGA dataset		ICGC dataset
	Training cohort	Testing cohort	Validation cohort
No. of samples	169	168	231
Age at diagnosis, years			
≤65	112	106	89
>65	57	62	142
Gender			
Female	52	55	61
Male	117	113	170
Grade			
G1-2	115	96	NA
G3-4	54	72	NA
TNM-stage			
StageI-II	127	123	141
StageIII-IV	42	45	90
T classification			
T1-2	128	125	NA
T3-4	41	43	NA

**FIGURE 2:**
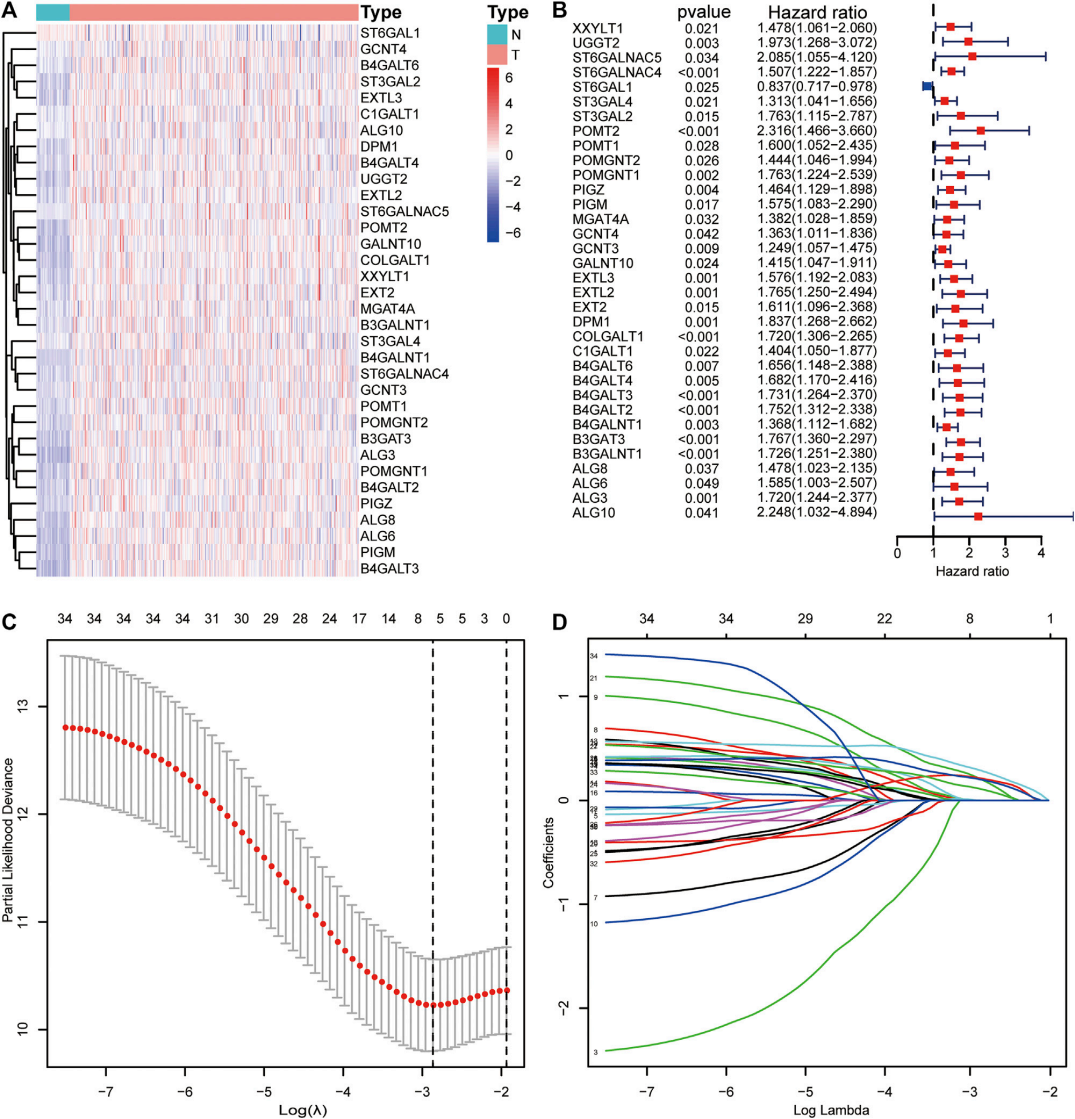
Visualization of candidate genes. **(A)** Heatmap of the expression levels of candidate genes. **(B)** Forest plot of candidate genes. **(C)** Partial likelihood deviance of different combinations of variables calculated via the LASSO Cox regression model. **(D)** LASSO coefficient profiles of candidate genes.

A total of 154 GT-related genes were generated by merging two gene sets, (Supplementary Table S2). Through initial analysis, 34 prognosis-related differentially expressed GTs overlapped as candidate genes for further analysis (Supplementary Table S3 and Figures 2A,B).

### Construction and Validation of Prognostic Signatures

Thirty-four candidate genes were used in LASSO regression analysis to confirm the core prognostic genes and to fit a risk prognosis model in the training cohort (*n* = 169). Finally, a prognostic risk score model comprising six genes (POMGNT1, DPM1, B4GALT3, B4GALT2, B4GALNT1, and B3GAT3) was constructed (Table 2 and Figures 2C,D). The following formula was utilized: risk score = (0.002*expression level of POMGNT1) + (0.231*expression level of DPM1) + (0.222*expression level of B4GALT3) + (0.122* expression level of B4GALT2) + (0.212* expression level of B4GALNT1) + (0.304*expression level of B3GAT3).

**TABLE 2:** Detail information of the prognostic gene signatures.

Gene symbol	Gene name	Lasso coefficient
POMGNT1	Protein O-linked mannose beta 1,2- N-acetylglucosaminyltransferase 1	0.00221148
DPM1	Dolichyl-phosphate mannosyltransferase polypeptide 1	0.23073783
B4GALT3	Beta-1,4-galactosyltransferase 3	0.22198892
B4GALT2	Beta-1,4-galactosyltransferase 2	0.12189376
B4GALNT1	Beta-1,4-N-acetyl-galactosaminyltransferase 1	0.21161694
B3GAT3	Beta-1,3-glucuronyltransferase 3	0.30439163

**FIGURE 3:**
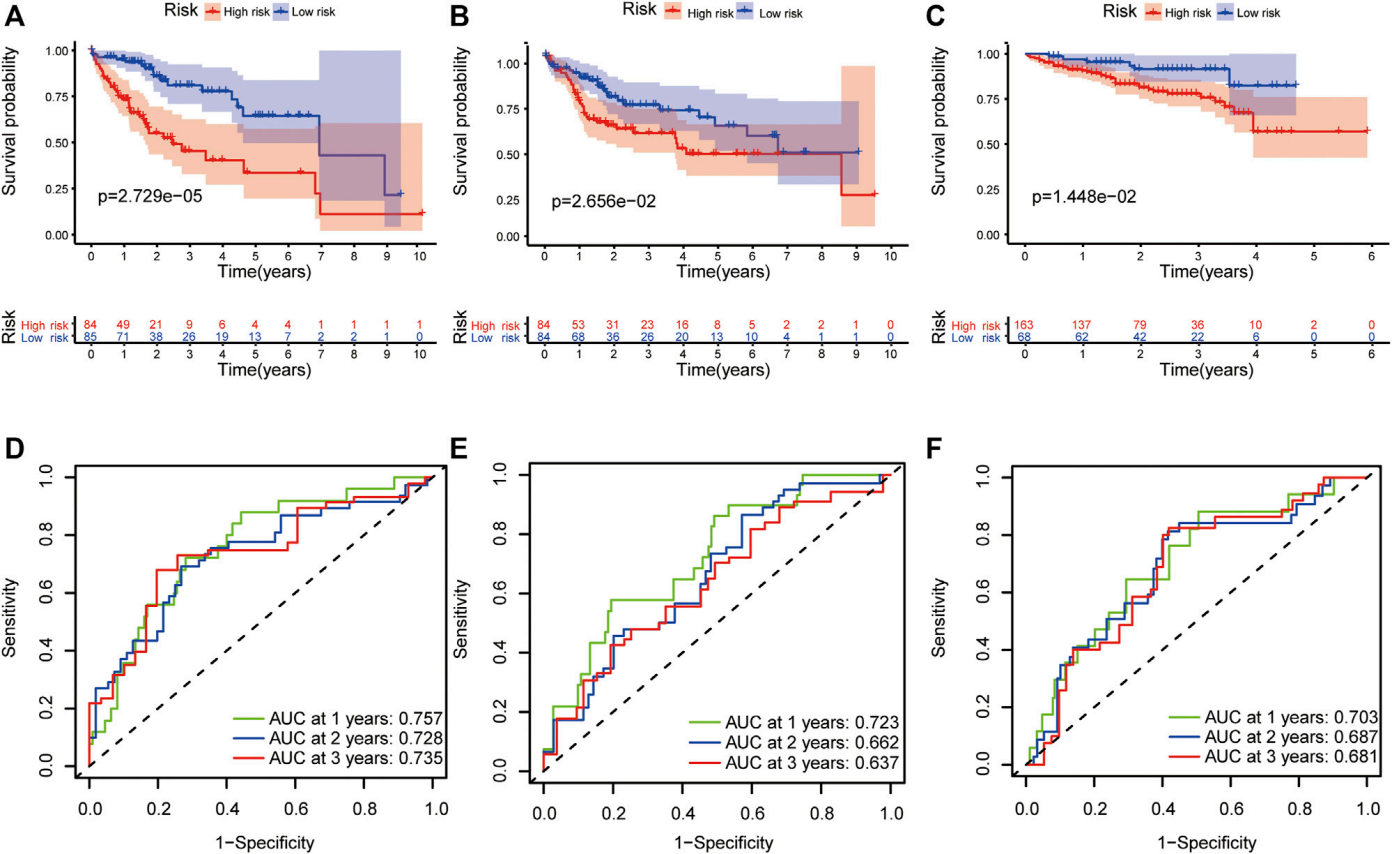
Validation of the prognostic signature. The Kaplan–Meier survival plots of high-risk and low-risk groups in the training cohort **(A)**, testing cohort **(B)** and validation cohort **(C)**. The ROC curves of the prognostic signature in 1-, 2-, and 3-years survival in the training cohort **(D)**, testing cohort **(E)** and validation cohort **(F)**.

**FIGURE 4:**
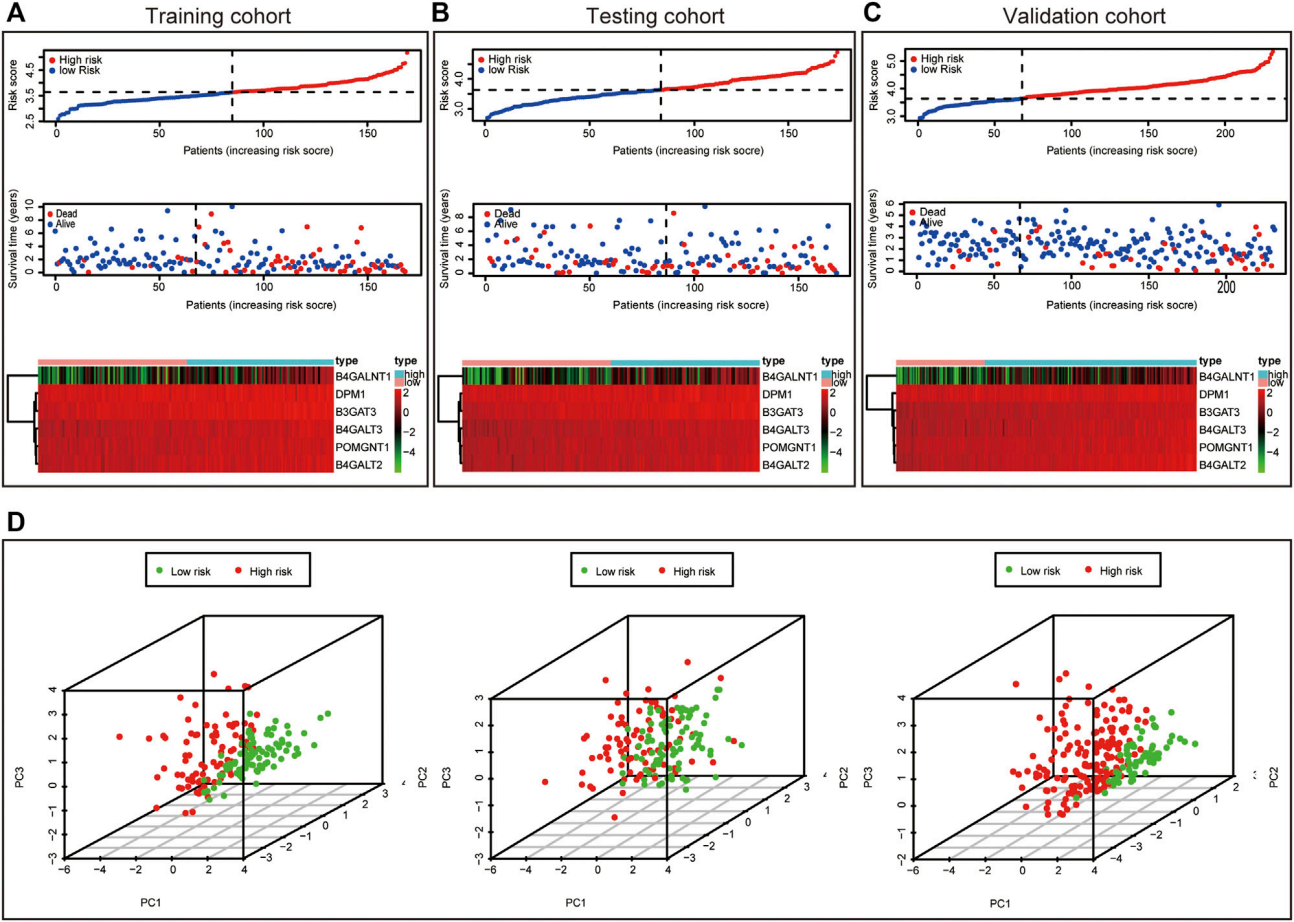
Characteristics of prognostic signature. Distribution of risk score, Survival status of HCC samples and Heat map of the expression of prognostic signature in the training cohort **(A)**, testing cohort **(B)** and validation cohort **(C)**. Principal component analysis (PCA) plot in the training cohort, testing cohort and validation cohort **(D)**.

This formula was used to evaluate outcomes in each sample and the optimal cut-off value for samples in the high-risk group and low-risk group was set at the median risk score in the training cohort. Kaplan-Meier analysis revealed that a significantly inferior OS was reflected in the high-risk group than in the low-risk group in the training cohort, testing cohort and validation cohort (Figures 3A–C). Then, ROC curves were plotted to verify how well the risk score predicted the risk of death at years 1, 2, and 3 (Figures 3D–F). In Figures 4A–C, the risk score curves, risk gene expression heatmap and patient survival status are shown based on the risk score values. Furthermore, principal component analysis (PCA) was implemented to visualize the sample information by risk group (Figure 4D). The results proved that the prognostic signature based on these 6 candidate genes had good predictive performance for HCC patients.

### Associations Between Risk Score and Clinicopathological Features

**FIGURE 5:**
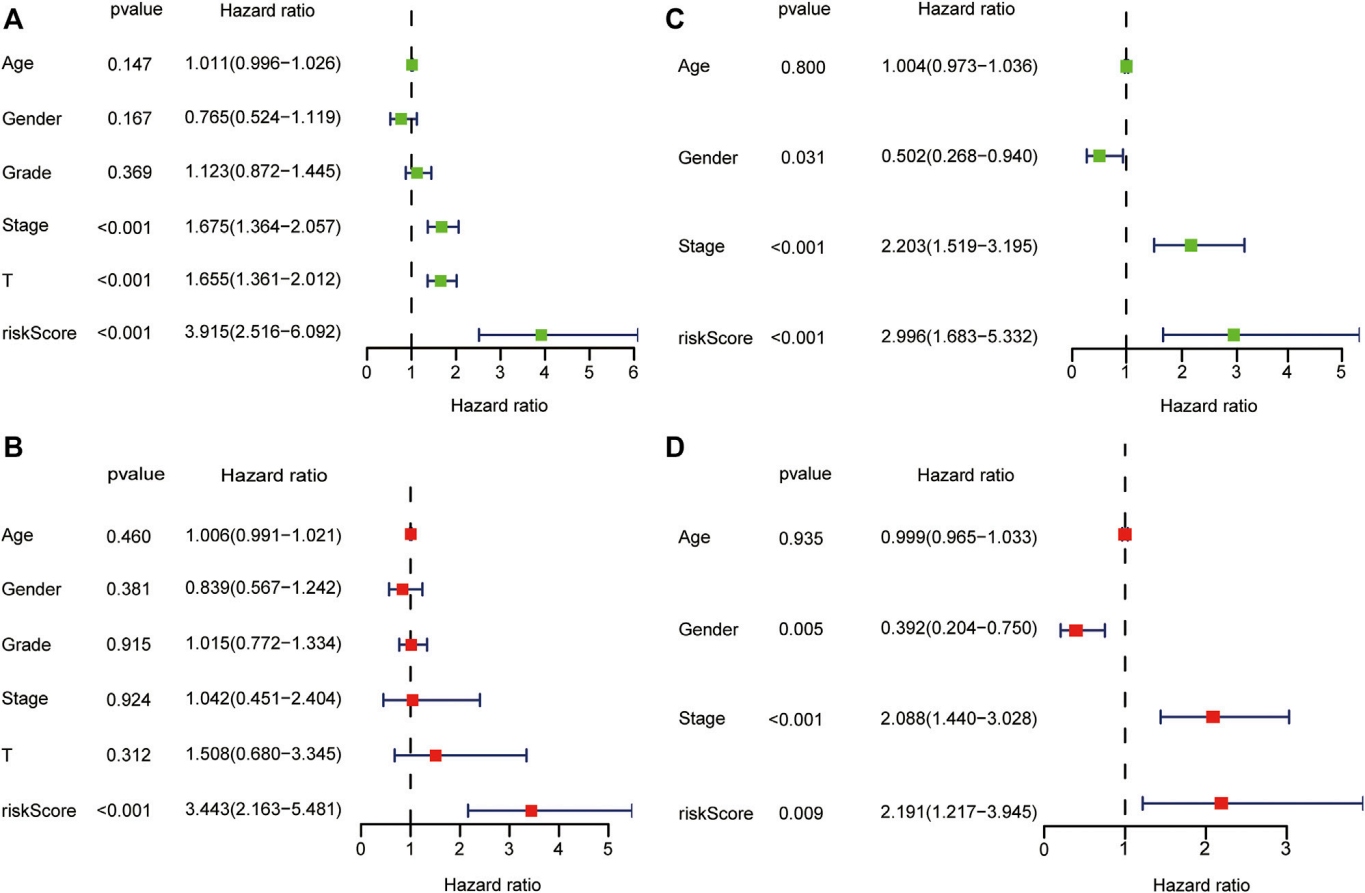
Forest plot of prognostic signature and clinical risk factors. The univariate Cox regression analysis in the TCGA dataset **(A)** and ICGC dataset **(C)**. The multivariate Cox regression analysis in the TCGA dataset **(B)** and ICGC dataset **(D)**.

In the TCGA dataset, the risk score was significantly associated with OS in univariate Cox regression analysis (HR = 3.915, 95% CI = 2.516–6.092, *p* < 0.001, Figure 5A). Likewise, multivariate analysis showed that the risk score was an independent prognostic indicator in HCC (HR = 3.443, 95% CI = 2.163–5.481, *p* < 0.001, Figure 5B). The results from the ICGC dataset were consistent with the above (Figures 5C,D).

**FIGURE 6:**
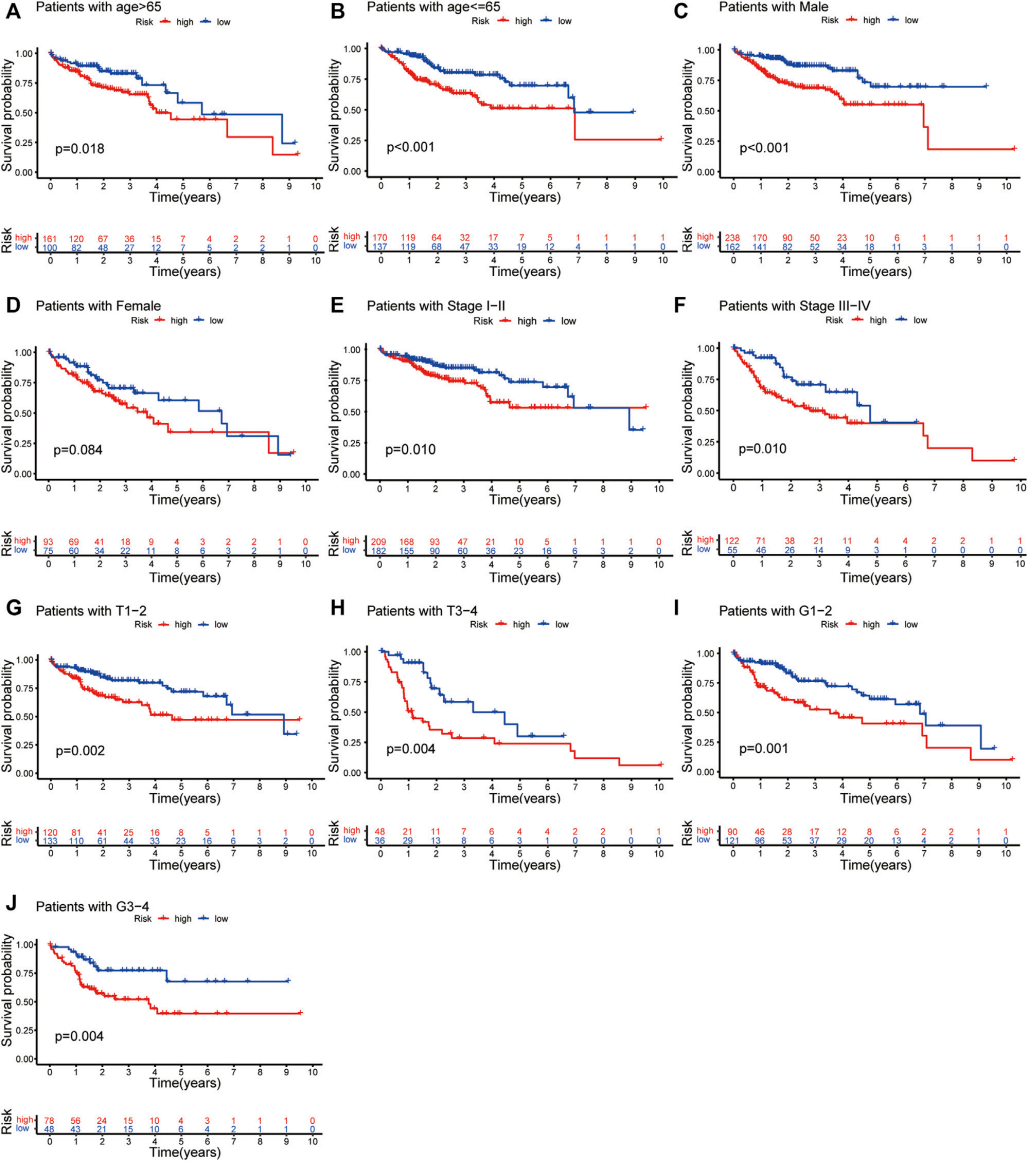
Independent prognostic analysis of risk scores and clinicopathological features. The Kaplan–Meier survival plots of patients with age >65 and ≤65 **(A,B)**; Males and females **(C,D)**; Stage I-II and Stage III-IV **(E,F)** in both TCGA and ICGC dataset. The Kaplan–Meier survival plots of patients with tumour stage T1-2 and T3-4 **(G,H)**; tumour grading G1-2 and G3-4 **(I,J)**.

For further analyses, we created prognostic subgroups of patients based on multiple classification approaches in both datasets. The results showed that OS between the two groups was significantly different in patients aged >65 years (Figure 6A, *p* = 0.018), ≤65 years (Figure 6B, *p* < 0.001), males (Figure 6C, *p* < 0.001), stage I-II (Figure 6E, *p* = 0.010) and stage III-IV (Figure 6F, *p* = 0.010). The difference in females did not reach significance (Figure 6D, *p* = 0.084). Furthermore, we used additional information from the TCGA to verify the above result (Figures 6G–J).

### The Construction of a Nomogram and Calibration Curve

**FIGURE 7:**
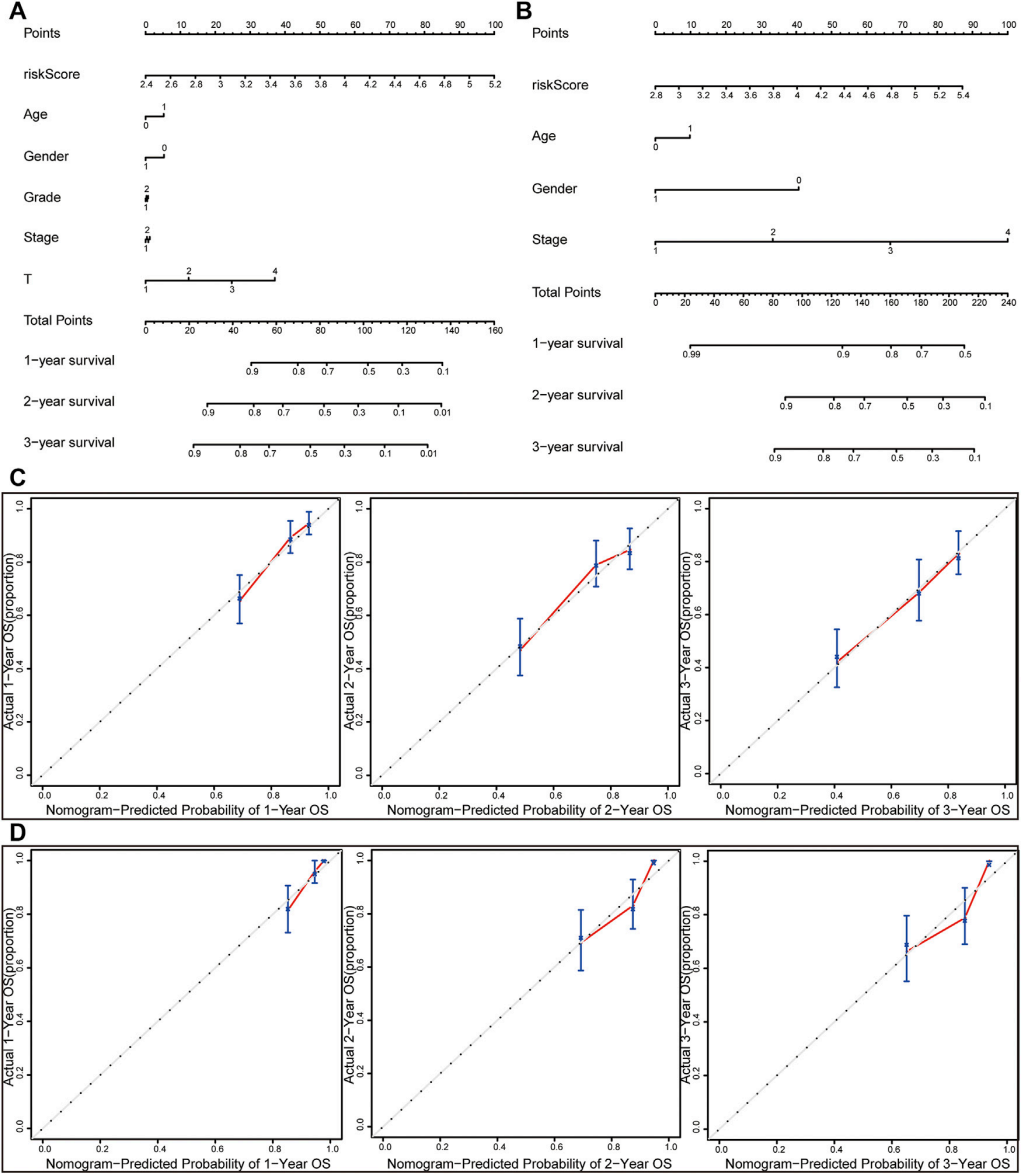
Nomograms and calibration curves for the prognostic signature. Nomograms for predicting the OS of 1-, 2-, and 3-years in the TCGA dataset **(A)** and ICGC dataset **(B)**. Calibration curves of nomograms in the TCGA dataset **(C)** and ICGC dataset **(D)**.

The nomogram was constructed by integrating the risk score with other clinicopathological features (Figures 7A,B). Furthermore, the calibration curve displayed linear concordance in the predicted and actual survival rates at 1, 2, and 3 years (Figures 7C,D). The findings suggested that the nomogram had high accuracy in predicting OS.

### Gene Set Enrichment Analysis

**FIGURE 8:**
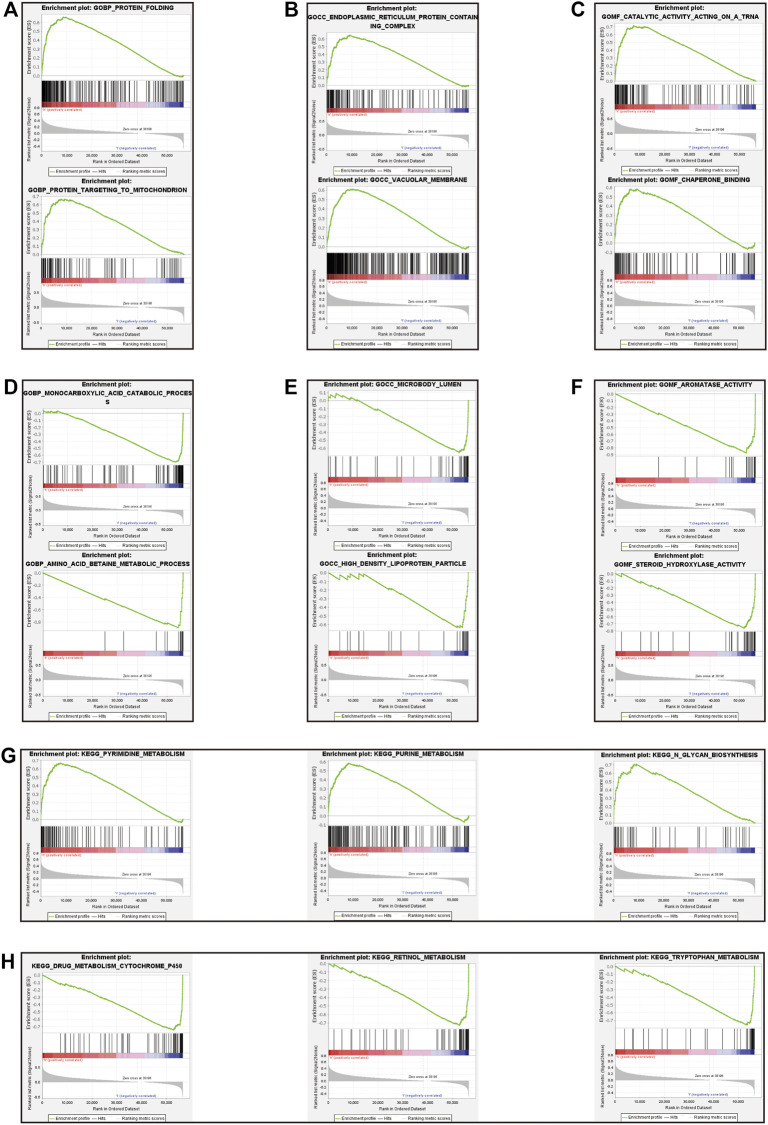
Gene set enrichment analysis between high-risk and low-risk groups. The result of top 3 in GO analysis in the high-risk group **(A–C)**. The result of top 3 in GO analysis in the low-risk group **(D–F)**. The upregulated KEGG pathways of top 3 in the high-risk group **(G)**. The upregulated KEGG pathways of top 3 in the low-risk group **(H)**.

Gene set enrichment analysis (GSEA) was done using Gene Ontology (GO) and Kyoto Encyclopedia of Genes and Genomes (KEGG). GO term analysis was used to evaluate the functional assessment of the different risk score groups, and the results demonstrated that the high-risk group was reportedly associated with protein folding, protein targeting to mitochondrion, endoplasmic reticulum protein containing complex, vacuolar membrane, catalytic activity acting on a tRNA and chaperone binding (Figures 8A–C). Additionally, monocarboxylic acid catabolic process, amino acid betaine metabolic process, microbody lumen, high density lipoprotein particle, aromatase activity and steroid hydroxylase activity were significantly downregulated in the low-risk group (Figures 8D–F). KEGG analysis showed that pyrimidine metabolism, purine metabolism, and N-glycan biosynthesis pathways were enriched in the high-risk group (Figure 8G); in contrast, some pathways in the low-risk group were enriched, including drug metabolism cytochrome P450, retinol metabolism and tryptophan metabolism (Figure 8H). We hypothesized that the prognostic signature may be potentially associated with metabolic disorders.

### Correlation of Risk Score With Tumour-Infiltrating Immune Cells

**FIGURE 9:**
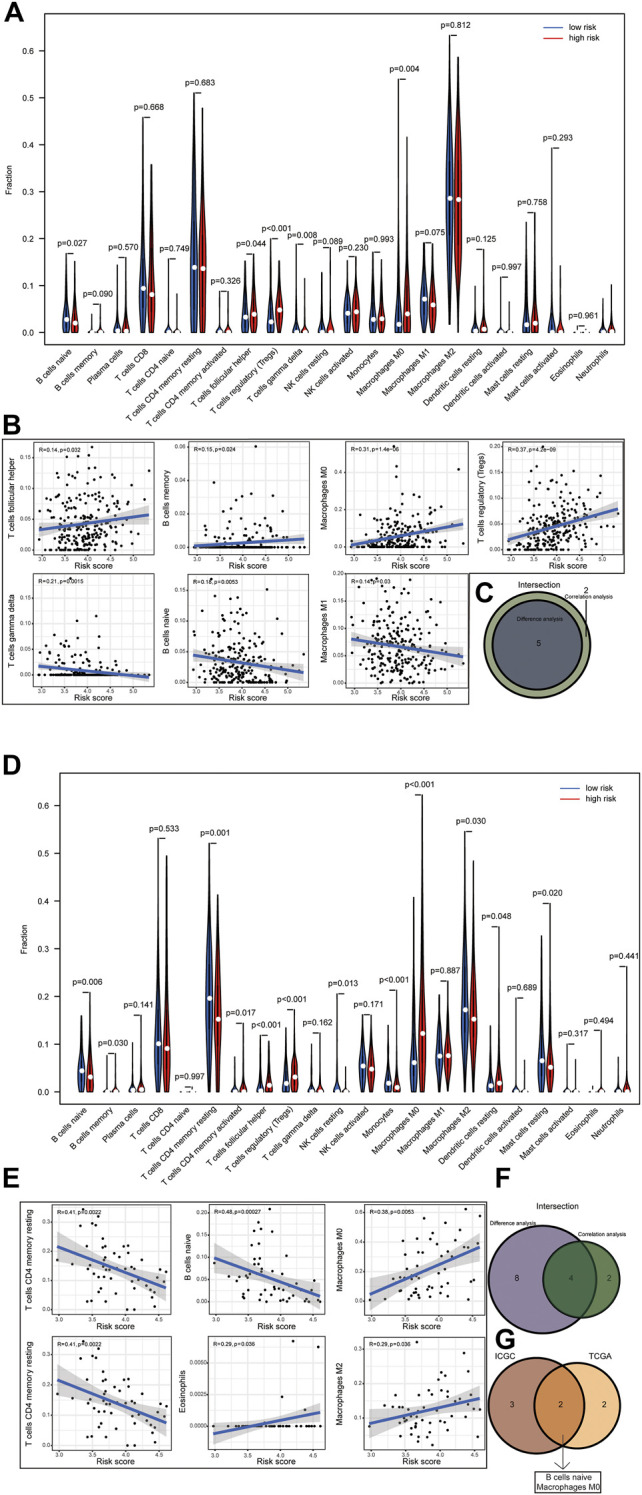
(1–2) Correlation of risk score with tumor-infiltrating immune. Results of the infiltrating level of 22 immune cell types in the ICGC dataset **(A)** and TCGA dataset **(D)**. Correlations of risk scores with immune infiltration level in the ICGC dataset **(B)** and TCGA dataset **(E)** (only significant correlations were plotted). Venn diagram of immune cells by the results of difference analysis and correlation analysis in the ICGC dataset **(C)** and TCGA dataset **(F)**. Result of the overlapping immune cell in the ICGC dataset and TCGA dataset **(G)**.

The relative abundance of 22 infiltrating immune cells was calculated by the CIBERSORT algorithm between the groups in both datasets. In the ICGC dataset, the infiltration levels of follicular helper T cells, regulatory T cells (Tregs) and M0 macrophages were higher in the high-risk group; however, naive B cells and gamma delta T cells were significantly enriched in the low-risk group; meanwhile, the correlation between immune cell infiltration and risk score was analysed (Figures 9A,B). Then, we observed higher levels of immune-infiltrating of M0 macrophages, regulatory T cells (Tregs), memory B cells, activated CD4 memory T cells, follicular helper T cells and resting dendritic cells in the TCGA’s high-risk group. In contrast, increased levels of naive B cells, resting CD4 memory T cells, resting NK cells, monocytes, M2 macrophages and resting Mast cells were found in the low-risk group. Similarly, we analysed the correlation between the risk score and TICS in the TCGA dataset (Figures 9D,E). By taking the intersection of results, the two most relevant TICS were identified as M0 macrophages and naive B cells (Figures 9C,F–G).

### Validating the Expression of Six Genes

**FIGURE 10:**
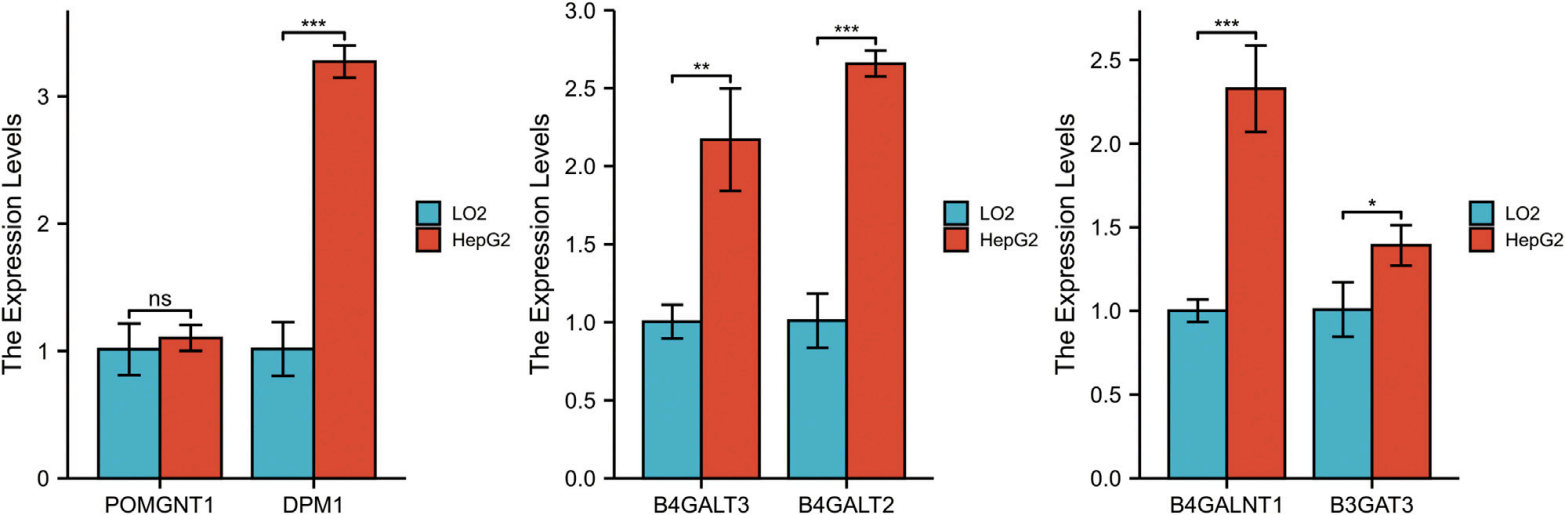
Validation of the mRNA expression levels of the prognostic genes in HCC cell line (HepG2) and normal hepatocyte cell line (LO2) using qRT–PCR.

**FIGURE 11:**
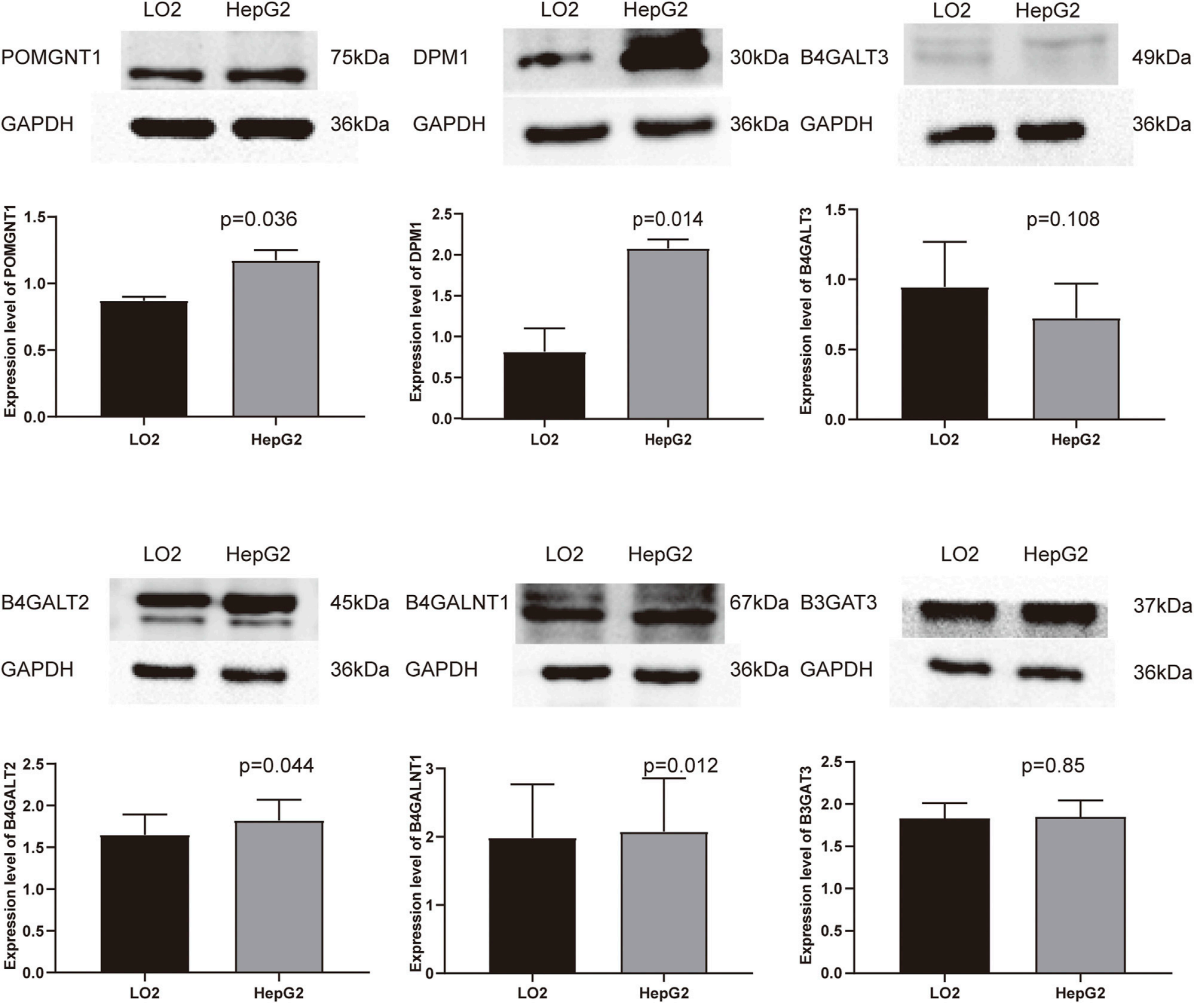
Validation of the protein expression levels of the prognostic genes in HCC cell line (HepG2) and normal hepatocyte cell line (LO2) using Western blot.

**FIGURE 12:**
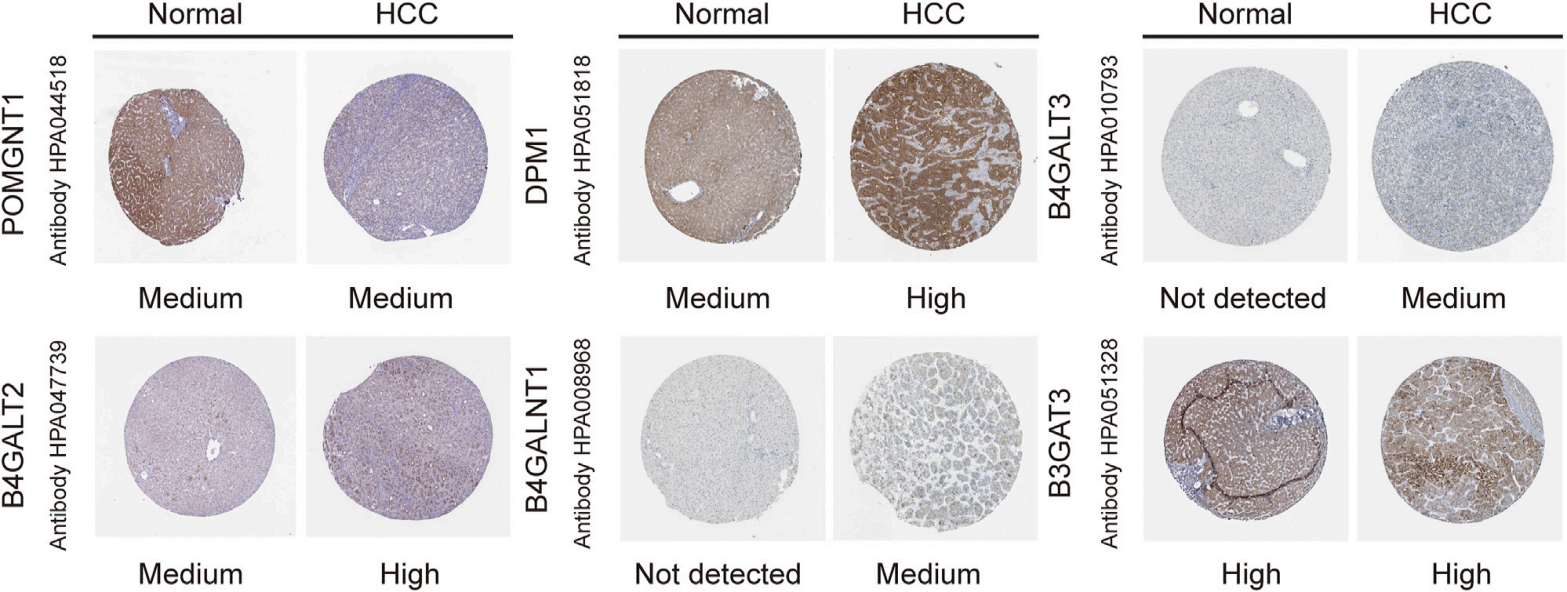
Immunohistochemistry staining of the prognostic genes in HCC and normal liver tissues derived from the HPA database.

To validate the differential expression at the mRNA level, we used qRT–PCR to compare the expression of the six genes in the HCC cell line (HepG2) and normal liver cells (LO2) (Figure 10). The mRNA levels of DPM1, B4GALT3, B4GALT2, B4GALNT1, and B3GAT3 were significantly higher in HepG2 cells than in LO2 cells. Subsequently, to validate the differential expression at the protein level, total cellular protein was analysed for the signature gene’s expression by western blot (Figure 11). Likewise, these six genes were compared in normal versus cancer tissues derived from the HPA, and the results are shown in Figure 12. Similar expression of POMGNT1 and B3GAT3 was observed in normal versus cancer tissues by immunohistochemical staining. However, the degree of staining in DPM1, B4GALT3, B4GALT2, and B4GALNT1 was stronger in cancer tissues than normal tissues.

## Discussions

Protein glycosylation, as the most common post-translational modification, plays an indispensable regulatory role in diverse biological functions (Pinho and Reis, 2015). Almost all proteins exert their functions through one or more of the 14 distinct glycosylation pathways (Schjoldager, et al., 2020). Given its critical role in tumour biology, aberrant glycosylation is regarded as a new hallmark of cancer (Munkley and Elliott, 2016), and offers a novel direction to predict cancer prognosis and treat cancer. In essence, aberrant glycosylation is due to dysregulation of GTs, and many of them are implicated in tumorigenesis as tumour suppressors or oncogenes. For instance, as a major metabolic integration point, OGT is upregulated in many tumours, including HCC, and it has been shown to be involved in the regulation of stem-like cell potential through modification of eIF4E (Cao, et al., 2019). Likewise, several studies have reported associations between dysregulated GTs and patient outcomes. Wu et al. detected the expression level of the sialyltransferase ST3GAL1 in 273 patients with HCC and found that upregulation of ST3GAL1 was an independent predictor of OS and disease-free survival (DFS) (Wu, et al., 2016). Liu et al. demonstrated that the polypeptide N-acetylgalactosaminyltransferase GALNT4 promoted the development of cancer as a tumour suppressor gene, and the level of expression could act as an independent favourable prognostic factor for recurrence-free survival (RFS) and OS (Liu Y. et al., 2017). In addition, integrating multiple genes could better predict the clinical outcome in the study of Kuo et al. (Kuo, et al., 2017). For this purpose, it is necessary to explore the prognostic signature of GTs for the accurate prediction of prognosis or response to therapy, which provides a reliable basis and reference for cancer management.

In this study, 34 prognosis-related differentially expressed GTs were first obtained. Then, LASSO regression was applied to construct a prognostic signature, as used in previous studies(Ueno, et al., 2021). The final screening result identified 6 genes (POMGNT1, DPM1, B4GALT3, B4GALT2, B4GALNT1, and B3GAT3); consistent with the screening results, we confirmed their differential expression in cells and tissues. The prognostic signature had strong robustness and stable prediction performance in the training and validation cohorts by a series of significance tests, and the risk score was identified as an independent prognostic indicator. Then, a nomogram comprising the risk score and clinicopathological data was generated to predict OS and showed superior performance in its validity. To gain more insight into the potential biological mechanism of the prognostic signature, we further used GSEA for the identification of biological functions. As expected, the results revealed that the prognostic signature was enriched in metabolism-related signalling pathways. Meanwhile, we know that glycosylation can modify protein structure and function; likewise, glycosylation affects immune cells with diverse functions and therefore modifies the tumour-immune microenvironment. M0 macrophages and naive B cells were identified as the most relevant TICS in our risk score group.

Coincidentally, the gene signature showed a tumour-promoting effect in our study. POMGNT1 has been examined in depth in glioblastoma (GBM), and it can promote proliferation and invasion by regulating EGFR/ERK signalling (Lan, et al., 2015); Furthermore, it induces temozolomide resistance of tumour cells in GBM by regulating the expression of factors in EMT signalling (Liu, et al., 2017b). In addition, it acts as a prognostic and predictive novel marker in GBM, similar to our results (Lan, et al., 2013). DPM1 acts as a core catalytic component of Dolichol phosphate mannose synthase (DPMS) (Tomita, et al., 1998). Li et al. reported that DPM1 serves as a biomarker for HCC patients’ prognostic prediction because the level of expression is significantly associated with clinicopathological parameters (Li, et al., 2020). The beta-1,4-1galactosyltransferase (B4GALT) family is a class of key enzymes that have crucial roles in many biological events, and catalyses the biosynthesis of N-acetyllactosamine on N-glycans by transferring UDP-galactose. It has been reported that upregulation of B4GALT2 induces p53-mediated apoptosis in HeLa cells and reveals a relationship with cisplatin-resistance in ovarian cancer cells (Zhou, et al., 2008; Zhao, et al., 2017). On the other hand, B4GALT3 has been evaluated more in-depth in tumour research than the former and it mainly plays a functional role by directly modifying β1-integrin glycosylation (Chen, et al., 2014; Chang, et al., 2013; Sun, et al., 2016). However, B3GALT3 develops different effects in different tumours, research shows that B4GALT3 overexpression can promote tumour growth and invasion in cervical cancer, neuroblastoma and GBM (Chang, et al., 2013; Sun, et al., 2016; Wu, et al., 2020), opposite to the tumour suppressor effects in colorectal and bladder cancer (Chen, et al., 2014; Liu, et al., 2018). In our study, we also found a high level of expression of this gene in HCC. B4GALNT1 (also known as GM2/GD2 synthase) functions as the key enzyme that transfers *N*-acetylgalactosamine (GalNAc) to GM3/GD3, catalysing the biosynthesis of gangliosides GM2/GD2 (Yoshida, et al., 2020). In breast cancer stem cells (CSCs), the upregulation of B4GALNT1 plays key roles in maintaining the CSC phenotype (Liang, et al., 2013). Jiang et al. confirmed that B4GALNT1 promoted the progression and metastasis of lung adenocarcinoma through the JNK/c-Jun/Slug signalling pathway and was involved in the tumour development of melanoma and clear cell renal cell carcinoma (Yoshida, et al., 2020; Jiang, et al., 2021; Yang, et al., 2019). B3GAT3 participates in the biosynthesis of the glycosaminoglycan (GAG) linker region of proteoglycan (PG) (Barré, et al., 2006). Given its important role in tumour metabolism, which was used repeatedly as a candidate gene for constructing prognostic models (Zhao, et al., 2021; Zhao, et al., 2020; Bingxiang, et al., 2021), its function of promoting the process of tumour EMT in HCC was confirmed by experimental verification (Zhang, et al., 2019). Based on the analysis above, the cancer-promoting effects of dysregulated expression are in accordance with our prediction results.

Risk prediction models have been developed as a powerful tool to provide references for clinical decision-making. A large amount of evidence has identified that dysregulation of GT expression plays critical roles in tumorigenesis, affecting the prognosis of HCC. For this reason, we developed a risk model and tried to explore its prognostic value. Although promising prediction results were displayed in our study, there is still room for improvement. First, we took a bioinformatics approach to mine GT data, which should be taken prudently and further validated by experimental studies before it is developed for clinical use. Second, to improve the efficiency of risk prediction in heterogeneity, further studies on larger sample sizes are needed.

## Conclusion

In summary, a computational risk model combining six GTs was developed to aid in the clinical prediction of HCC prognosis. The model showed good prediction efficiency after verification by the internal testing and the external validation cohort. Furthermore, these prognostic markers were validated by western blot and qRT–PCR. However, further studies should be conducted to explore the clinical value of our current study.

## Data Availability

The original contributions presented in the study are included in the article/Supplementary Material, further inquiries can be directed to the corresponding authors.
